# Study protocol: optimization of complex palliative care at home via telemedicine. A cluster randomized controlled trial

**DOI:** 10.1186/1472-684X-10-13

**Published:** 2011-08-09

**Authors:** Froukje Duursma, Henk J Schers, Kris CP Vissers, Jeroen Hasselaar

**Affiliations:** 1 Department of Anesthesiology, Pain and Palliative Medicine, Radboud University Nijmegen Medical Centre, PO Box 9101, 6500 HB Nijmegen, The Netherlands; 2 Department of Primary and Community Care, Radboud University Nijmegen Medical Centre, PO Box 9101, 6500 HB Nijmegen, The Netherlands

## Abstract

**Background:**

Due to the growing number of elderly with advanced chronic conditions, healthcare services will come under increasing pressure. Teleconsultation is an innovative approach to deliver quality of care for palliative patients at home. Quantitative studies assessing the effect of teleconsultation on clinical outcomes are scarce. The aim of this present study is to investigate the effectiveness of teleconsultation in complex palliative homecare.

**Methods/Design:**

During a 2-year recruitment period, GPs are invited to participate in this cluster randomized controlled trial. When a GP refers an eligible patient for the study, the GP is randomized to the intervention group or the control group. Patients in the intervention group have a weekly teleconsultation with a nurse practitioner and/or a physician of the palliative consultation team. The nurse practitioner, in cooperation with the palliative care specialist of the palliative consultation team, advises the GP on treatment policy of the patient. The primary outcome of patient symptom burden is assessed at baseline and weekly using the Edmonton Symptom Assessment Scale (ESAS) and at baseline and every four weeks using the Hospital Anxiety and Depression Scale (HADS). Secondary outcomes are self-perceived burden from informal care (EDIZ), patient experienced continuity of medical care (NCQ), patient and caregiver satisfaction with the teleconsultation (PSQ), the experienced problems and needs in palliative care (PNPC-sv) and the number of hospital admissions.

**Discussion:**

This is one of the first randomized controlled trials in palliative telecare. Our data will verify whether telemedicine positively affects palliative homecare.

**Trial registration:**

The Netherlands National Trial Register NTR2817

## Background

Palliative care has become an important public health issue since the past decade [1]. The ageing of the population and the rising life expectancy are contributing to this development. Also, the pattern of diseases people suffer and die from has changed from acute illnesses towards chronic illnesses [1-3]. In addition to advances in medical knowledge and technology that increase treatment possibilities at the end of life, these epidemiological transitions have led to a growing need of palliative care in the last phase of life [4].

The primary goal of palliative care is to ensure the best possible quality of life of patients and their families facing a life threatening illness [1,5]. Most people in their end-stage of life, regardless of their initial disease, want to be cared for and to die at home [6,7]. Therefore, place of death is considered an indicator of quality of end-of-life care [8]. However, research in Belgium and in the Netherlands has shown that 30-40% of palliative patients are transferred from home to a hospital or health care institution in the last week of life [9,10]. This trend is also seen internationally [11]. Transitions in the location of care are often extremely stressful for patient and caregivers [11] and can pose a challenge for the continuity of care [11,12].

Place of death has also become a topic of wider interest for public health policy, due to the focus in health care on cutting costs in acute care settings [13]. Many European countries have implemented policy measures to reduce the number of acute care hospital beds as a means to restrict hospital expenditure [5]. With this shift in location of care for the seriously ill from hospital to home, the reliance on family caregivers to support patients with terminal illness at home is growing [13]. These family caregivers are of vital importance for those wanting to die at home. Without them, remaining at home in the last phase of life would be impossible for many patients [14,15]. However, caregiving for terminally ill patients can be burdensome for informal caregivers, possibly leading to burn-out [16,17].

Due to a growing number of palliative patients and the desire for less institutionalized care, community-based palliative care will become a big challenge [18]. The development of innovative approaches to deliver good quality of care at home is therefore necessary. One such approach is the use of telemedicine. Telemedicine is the use of telecommunications and information technologies to share and maintain patient health information and to provide clinical care and health education to patients and professionals when distance separates the patients [19]. Teledermatologic consultation has been one of the first applications of telemedicine. Literature shows that this form of teleconsultation reduces the number of traditional face-to-face consultations with a dermatologist [20-23]. In addition, a telemedicine approach has also been proven cost-effective in diabetes care and pediatrics [24,25]. In the field of palliative homecare however, few quantitative studies have been carried out [26-30]. These studies often are of moderate methodological quality. Common shortcomings are small sample sizes, comparability of intervention and control groups and the handling of drop-outs [26,31].

Teleconsultation is a specialized form of telemedicine that uses technology to provide real-time visual and audio patient assessment [32]. Teleconsultation is an instrument to bring across expertise from the hospital into primary healthcare and can therefore be very useful in complex homecare for palliative patients and their families. This study aims to evaluate the effectiveness of teleconsultation in palliative homecare. The primary goal is to evaluate the effectiveness of teleconsultation on the symptom burden of palliative patients at home. Secondary objectives are 1) to investigate whether teleconsultation influences the number of hospital admissions by acting more pro-active on escalating problems of patients, 2) to consider if the burden of the family caregiver changes by giving them a better opportunity to address their needs and problems, 3) to study the patient experienced continuity of medical care in the last phase of life, 4) to assess patient and caregiver satisfaction with the teleconsultation contact and 5) to investigate patient's problems and needs for palliative care.

The objective of this report is to present the protocol of the study used for data collection in 2011 and 2012.

## Methods/Design

### Study design

The study consists of a two-armed cluster randomized controlled trial. To prevent possible bias at the level of GPs, a clustering will take place on the level of the GP. The symptom burden of the patient and the secondary outcomes in the two study arms will be compared.

Parallel to this cluster randomized controlled trial, a qualitative study will be undertaken. In this qualitative study, semistructured interviews and observations will be used to consider the socio-ethical aspects of teleconsultations in palliative homecare. The findings from the quantitative and the qualitative study will be integrated in future articles.

### Randomization

Participating GPs will be randomly assigned to the intervention group or to the control group. Due to clustering of GPs, all subsequently referred patients will be in the same study group. A block design with different length of blocks (4 and 6) will be used to give an equal balance between groups. An independent researcher will generate and store the randomization code.

### Study population

On the moment of inclusion, the patient's GP acts as the coordinator of medical care, and patients reside at their homes. Besides the place of residence, the inclusion criteria are:

- Dutch-speaking patients, aged 18 years or older, with a progressive oncological disease,

- a score of ≤ 60 on the Karnofsky Performance Scale (assessed by the GP),

- a life expectancy of ≤ 3 months.

Patients unable to give informed consent and patients with an active psychotic disorder or a serious cognitive disorder are not eligible for inclusion.

### Intervention

After completing the baseline measurement, a telemedicine computer will be installed at the patient's home. Soon after the installation, the nurse practitioner of the consultation team contacts the patient to make an appointment for the first teleconsultation. During this first digital screen-to-screen contact between the patient and the nurse practitioner, the nurse checks for problems in palliative care following a predefined consultation protocol (e.g. physical problems, social problems, coordination of care). After the first teleconsultation, the nurse practitioner, in cooperation with the palliative care specialist of the palliative consultation team, advises the GP on the treatment policy for the patient. During this trajectory, the GP continues to be the coordinator of medical care. The teleconsultations will return every week, but more frequent contact is possible when the patient and the team desire this. There are no installation or internet costs for the patient and also the use of the telemedicine computer is for free.

The telemedicine application is a computer with a touch screen, a microphone/speaker and a camera. Large and easy to understand pictograms make the program user-friendly. To contact a nurse or a physician for a videoconference, the patient just has to touch the image of that person. In addition to the weekly teleconsultations, the patient also has the opportunity to videophone the 24/7 support service of the homecare organization. Furthermore, an information database, an internet-browser and some entertainment options are available on the telemedicine application. The telemedicine application will not be used in emergency situations due to safety restrictions.

### Collaborating organizations

This research protocol is granted by The Netherlands Organisation for Scientific Research (NWO). The project is coordinated by the Department of Anesthesiology, Pain and Palliative Medicine of the Radboud University Nijmegen Medical Centre. This department works in close collaboration with the Department of Primary and Community Care. ZZG Zorggroep, a regional homecare organization, provides the patients with a 24/7 support service. Finally, an ICT-installation company (FocusCura) installs the telemedicine application at the patient's home and also provides technical support.

### Recruitment, consent and data collection

For the purpose of this study, all GPs practicing in the Nijmegen region are invited with a letter, in which the aim and procedure of the study are clarified. Furthermore, the advantages and disadvantages of participating are mentioned and contact information for advice from an independent physician is given. The researcher contacts the GPs several days after receipt of the letter. When more information is needed, the researcher visits the GP to inform him/her more extensively about the trial. When a GP refuses to participate, the researcher will document the arguments for non-respondent analysis. Inclusion starts at April 1 2011 and runs to October 1 2012.

**Figure 1:**
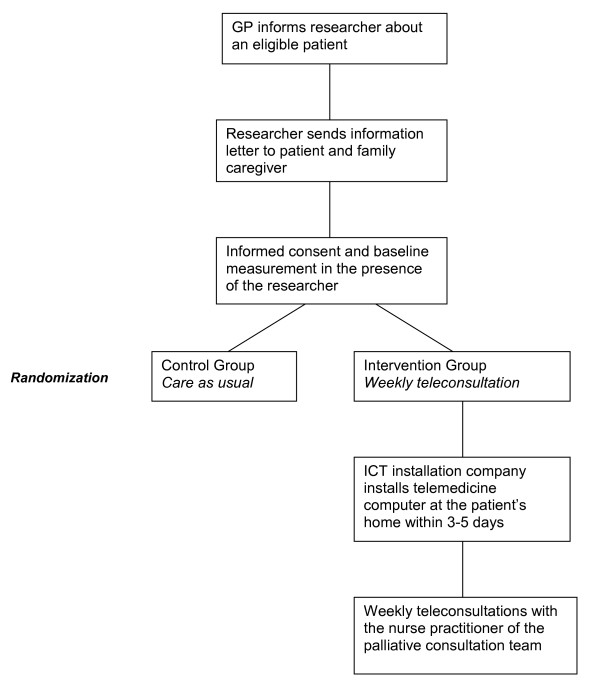
**Flowchart of the inclusion**.

When the patient and the family caregiver decide to join the study, they sign the informed consent form during a one-hour home visit by the researcher. After informed consent, the patient and the family caregiver will first complete the baseline measurement. The baseline measurement consists of several demographic questions and four short questionnaires for the patient. The four questionnaires (ESAS, PNPC-sv, HADS and NCQ) will be completed at home every four weeks during the study participation. The ESAS will be completed every week, as symptom burden is our primary outcome. The family caregiver completes a questionnaire on self-perceived pressure from informal care (EDIZ) at baseline and every two weeks. At time points where there is no home visit, the ESAS and EDIZ will be returned in a stamped envelope. The family caregiver receives a mobile phone text message as a reminder to fill in and post the questionnaires. The flowchart of the inclusion is described in Figure 1.

### Outcome measures

#### Primary outcome

The symptom burden experienced by the patient, using the Edmonton Symptom Assessment System (ESAS) and the Hospital Anxiety and Depression Scale (HADS).

#### Secondary outcomes

1. The number of hospital admissions, which will be obtained from the patient's file.

2. The experienced problems and needs for palliative care (PNPC-sv; Problems and Needs in Palliative Care).

3. Patient and caregiver satisfaction with the teleconsultation (PSQ; Patient Satisfaction Questionnaire).

4. The experienced continuity of medical care in the last phase of life (Nijmegen Continuity Questionnaire; NCQ).

5. The experienced burden of the family caregiver (EDIZ; self-perceived pressure from informal care).

#### Other study outcomes

1. We will ask for some demographic information, such as age, marital status, number of children and living situation.

2. After the period of study inclusion (from the GPs patient record):

• Number of contacts by telephone with the GP practice

• Number of home visits by the GP

• Number of contacts with the GPs out of hours service

• Number of patients with complex interventions (such as palliative sedation)

• Number of and indications for hospital admissions

• Date and place of death

### Measurement instruments

The vulnerable condition of the patients was an important point of departure in the selection of the questionnaires. Important issues considered were length of the questionnaire, difficulty, the number of questionnaires and the time points of measurement. When it is impossible for the patient to complete the questionnaires independently, the informal caregiver is allowed to assist the patient. Table 1 gives an overview of the questionnaires used in the study.

**Table 1:** Questionnaires used in the study

**Patient questionnaires**
Administered at baseline
• Basic demographic information (7 questions)
Administered at baseline and every week
• **ESAS **(Edmonton Symptom Assessment System)
- 10 items on symptom assessment
Administered at baseline and every four weeks
• **PNPC-sv **(Problems and Needs in Palliative Care - short version)
- 33 questions on experienced problems and needs for care
• **HADS **(Hospital Anxiety and Depression Scale)
- 14 items on anxiety (7 items) and depression (7 items)
• **NCQ **(Nijmegen Continuity Questionnaire)
- 28 items within 3 subscales on continuity of care
**Family caregiver questionnaire**
Administered at baseline and every two weeks
• **EDIZ **(one dimensional assessment of care burden)
- 9 items on the experienced burden from informal care
**Patient, GP and a member of the palliative consultation team**
Administered after the first two teleconsultations
• **PSQ **(Patient Satisfaction Questionnaire)
- 5 questions on satisfaction with the teleconsultation

#### Primary outcome

The ESAS is an easy to complete questionnaire developed for use in daily symptom assessment of palliative care patients. The patient rates the presence and severity of the following nine symptoms common in cancer patients: pain, activity, nausea, depression, anxiety, drowsiness, appetite, sense of well-being and shortness of breath. An optional tenth symptom can be added by the patient [33]. The items are rated on 0-10 visual numerical scales (with 10 being the worst imaginable intensity of a symptom). The ESAS is widely used and proven to be reliable [34,35].

The HADS is a 14-item self-report screening scale that was originally developed to indicate the possible presence of anxiety and depressive states in the setting of a medical non-psychiatric out-patient clinic. It contains two 7-item subscales on anxiety and depression. Each item scores on a 4-point Likert scale. The questionnaire assesses symptoms over the preceding week [36]. Psychometric properties of the HADS were assessed in six different groups of Dutch subjects (*N *= 6165). Homogeneity and test-retest reliability of the total scale and the subscales were considered adequate. The dimensional structure and reliability of the HADS is considered to be stable across medical settings and age groups [37].

#### Secondary outcomes

The PNPC is a self-reporting questionnaire for patients covering all dimensions of palliative care to investigate their problems and (unmet) palliative care needs. Experienced problems and needs for care are addressed separately, because patients could have had adequate assistance despite enduring symptom suffering. The original questionnaire with its 90 items has shown validity and reliability, but is not always practical for palliative patients. Therefore, a short version of 33 items has been developed and validated. This PNPC- short version was tested on 94 patients with metastatic cancer and has demonstrated construct validity. The dimension reliability was satisfactory, although two domains were less coherent [38].

The PSQ is a 5-item visual-analogue screening tool to measure patients' satisfaction, as well as doctor's satisfaction, following the consultation [39]. The questionnaire is developed and tested in the home situation in the Netherlands. Physician satisfaction turned out to be substantially lower than patient satisfaction, both at item level and at overall satisfaction level [39]. This finding is consistent with other patient satisfaction studies [40,41]. The PSQ is a short and easy to fill in questionnaire compared to many other patient satisfaction questionnaires and is therefore a very suitable instrument for the vulnerable patients participating in this study. The questionnaire will be completed by the patient, a member of the palliative consultation team and the GP following the first two teleconsultations.

The NCQ is developed and validated by the Radboud University Nijmegen Medical Centre (Department of Primary and Community Care). The questionnaire measures the patients' experienced continuity of care across primary and secondary care settings and consists of 3 subscales: 'Personal or relational continuity: care provider knows me' (5 items), 'Personal continuity: care provider shows commitment' (3 items) and 'Team/cross-boundary continuity' (4 items). Items are scored on a 5-point Likert scale, with an additional option to choose '?' ('do not know'). The NCQ was tested on 268 patients with a chronic disease and proved to be a reliable and valid instrument with good discriminant abilities [Uijen AA, Schellevis FG, Mokkink HGA, van Weel C, van den Bosch WJHM, Schers HJ: Measuring continuity from the patient perspective: psychometric properties of the Nijmegen Continuity Questionnaire (NCQ), submitted]. In this study, only the domains on the experienced quality of the relation between GP and specialist and the confidence in the GP and in the specialist are being used. Publications on the development of the questionnaire and the examination of the reliability and validity have been submitted to a journal and are available on request.

The EDIZ is a 5-point Likert scale screening questionnaire with 9 subjects to measure the self-perceived burden from informal care. This burden is expressed in thoughts (e.g. 'the situation of my.... is constantly on my mind') as well as in his/her interaction with the social environment (e.g. 'it's not easy to combine the responsibility for my ... with the responsibility for my work/family'). The EDIZ is a validated instrument [42].

### Sample size calculation

The null hypothesis of this cluster randomized trial is that there are no significant differences in symptom distress between palliative patients at home with and without a telemedicine-computer for videoconference. Symptom distress will be measured by the Edmonton Symptom Assessment Scale (ESAS). The ESAS is a 0 to 10 numeric scale (0 = best, 10 = worst) to rate severity of 10 symptoms. The sum of all 10 scales makes the Total Distress score (max.100). Based on a study of Follwell et al. [43] we determined a Total Distress score of 8 as the minimum clinically important difference for the power calculation. Without a cluster-effect and without repeated measures, we would need 80 patients per condition, assuming an α of .05 and a power of 80% (calculated with nQuery advisor 4.0). However, there is a cluster-effect and there are repeated measures that we corrected for.

To correct for clustering, we multiplied the abovementioned sample size with the design factor (1+(n-1)ICC), where n is the number of patients and ICC the intra cluster correlation. Assuming 3 enrolled patients per GP and an ICC of 0,1 (based on Knox & Chondros [44]), the design factor for clustering is 1,2.

To correct for repeated measures and baseline measurement, we also multiplied the sample size with the design factor ((1+(k-1)ρ)/k- ρ_0_^2^). In this formula, k is the number of repeated measures, ρ is the (mean) correlation between pairs of post-tests and ρ_0 _is the (mean) correlation between a post-test and the baseline measurement. Here, k = 13, and we state ρ = 0,45 [34] and ρ_0 _= 0,35. The design factor for repeated measures is therefore 0,37.

That makes the total required sample size 80 × 1,2 × 0,37 = 36 patients per condition. Taking into account an early drop-out of patients, we aim to include 50 patients per condition. This calculation is based on Follwell et al. [43], who considered a drop-out of 30% within an inclusion period of 1 month. Because the inclusion period in our study is longer (estimated life-expectancy of approximately 3 months at inclusion), we chose a higher drop-out percentage (39%).

Put briefly, a sample size of 100 patients (α = .05, power = 80%) is required to detect differences in change of symptom distress between the intervention group and the control group.

### Statistical analysis

The data will be stored and analyzed in the Radboud University Nijmegen Medical Centre using the Statistical Package for the Social Sciences (SPSS version 16.0, SPSS inc., Chicago, Illinois, USA). Data cleaning will be performed via SPSS syntax operations. All statistical tests will be done two-tailed with 95% confidence intervals.

#### Descriptive statistics

Normally distributed quantitative data will be analyzed by mean and standard deviation. Data that are not normally distributed will be reported by median and interquartile range. Qualitative data will be reported by frequency distributions and percentages.

#### Multivariate analysis

Our primary goal is to detect differences in the ESAS and HADS-scores between groups of patients with and without the telemedicine application. Because the study design involves a pretest, repeated measures and clustering, data will be analyzed with Linear Mixed Models. This method of analysis will also be used to describe our secondary outcome measures (EDIZ, NCQ, PNPC-sv, PSQ, number of hospital admission).

### Ethical considerations

Actively participating in the teleconsultations and completing the questionnaires can be burdensome for this vulnerable group of patients, particularly towards the end of the study period when the condition of the patient worsens. Therefore, the researcher, the GP and the palliative consultation team always take into account the condition of the patient when a research activity will be undertaken. The disadvantages of participating, as well as the advantages, are clearly mentioned in the information letter to patient and informal caregiver.

The protocol of the present study was approved by the Central Committee on Research Involving Human Subjects (CCMO) Arnhem/Nijmegen.

## Discussion

This study investigates the effectiveness of teleconsultation in complex palliative homecare. It compares clinical outcomes in the intervention group with a control group. The intervention consists of a weekly teleconsultation with the palliative consultation team. Bringing specialist expertise to the home via video-telephone technology is an innovative way of improving complex homecare for palliative patients. A strength of our study is the robust design. We plan to conduct a cluster randomized controlled trial, which will be one of the first in palliative homecare, at least in the field of telemedicine. Furthermore, symptom burden is our primary outcome measure. Studies with clinical outcome measures are scarce in research on palliative homecare. Therefore, future data on this primary outcome measure, when positive, will be very helpful in the adoption and implementation of telemedicine services in palliative care.

However, there are also several challenges in this study. A first challenge will be to enroll a sufficiently large sample to make sure that differences between the intervention group and the control group can be detected. If recruitment problems occur, the palliative consultation team and the regional home care organization will additionally be involved. Finally, this research project stimulates collaboration between primary care and hospital care in order to optimize the continuity of care. Besides this process innovation, we also focus on technical/product innovation. In a world where technology is changing rapidly, it is a big challenge to carry out innovative research.

## Competing interests

The authors declare that they have no competing interests.

## Authors' contributions

KV, HS and JH contributed to the development and the design of the protocol. JH and KV developed the analysis plan and applied for funding. FD has drafted the manuscript with critical input from all other authors who have read and approved the final manuscript.

## Pre-publication history

The pre-publication history for this paper can be accessed here:

http://www.biomedcentral.com/1472-684X/10/13/prepub
